# Gingival lymphatic malformation. An atypical case report

**DOI:** 10.4317/jced.60488

**Published:** 2023-06-01

**Authors:** Javier Alberdi-Navarro, Nerea Ruiz-Ochoa, Andoni De Juan-Galindez, Agurne Uribarri-Etxebarria

**Affiliations:** 1 DDS, MS Oral Pathology, PhD. Dental Clinic Service. Department of Stomatology II. School of Medicine and Nursing. University of the Basque Country (UPV/EHU). Leioa, Spain; 2 DDS. Department of Stomatology II. School of Medicine and Nursing. University of the Basque Country (UPV/EHU). Leioa, Spain; 3 DDS, MD, PhD. Euskalduna Clinic. Bilbao. Spain; 4 DDS, MS Oral Surgery and Implantology, PhD. Dental Clinic Service. Departament of Stomatology I. School of Medicine and Nursing. University of the Basque Country (UPV/EHU). Leioa, Spain

## Abstract

Lymphatic malformations are a rare pathology that presents a highly variable clinical expression. Intraorally, it mainly affects the dorsum of the tongue. The objective of this work is to present a case of lymphatic malformation in an atypical location. A 20-year-old male who attended the clinic for multiple vesicular lesion in attached gingiva, asymptomatic and of unknown evolution. Removal of the lesion and subsequent histological analysis were performed, which showed a microcystic lymphatic vascular lesion. Immunohistochemistry for D2-40 was performed, which corroborated the lymphatic origin of the lesion. At 6 months, no recurrence of the lesion was recognized. Clinicians should include lymphatic malformations in the differential diagnosis of multiple vesicular lesions. Knowing the oral manifestations of this entity is essential for its proper diagnosis and clinical management.

** Key words:**Gingiva, oral lymphatic malformation, diagnosis.

## Introduction

Lymphatic malformations (LM) or lymphangiomas are an uncommon pathology, characteristically affecting the body areas that are richest in lymphoid tissue, including the head and neck region, armpits and mediastinum (1,2). Specifically, the head and neck region, including the oral cavity, is the area with the highest prevalence, since between 45 and 52% of lymphatic malformations in the body develop in this area (1).

Lymphatic malformations are currently considered a congenital developmental disorder, rather than a neoplasm per se (3,4). In fact, most cases are detected at birth or in the first 3 years of life (1). Most of the cases show growth associated with body development, although there are factors that may cause lesions to grow, such as mechanical trauma, infections or hormonal alterations.

From a structural point of view, lymphatic malformations can be classified as microcystic, macrocystic or mixed (3,5). At intraoral level, most lesions are microcystic and mainly affect the tongue, more specifically the dorsum of the tongue. Other locations that may be affected are the lips, buccal mucosa, or palate (5).

Clinical manifestations will depend on the type of lymphatic malformation (macro/microcystic), its depth and location. In the oral cavity, they usually appear as clusters of vesicular, raised, soft and depressible lesions, usually of the same colour as the mucosa or with a yellowish or even erythematous appearance, due to hematic extravasation. Although it is true that, in cases of deep lesions, they may present as a diffuse increase in volume (1).

The diagnosis of this entity is eminently clinical, and histopathological study is necessary for its confirmation. Occasionally and especially in the case of deep and extensive lesions, imaging tests are required to assess the extent of the lesion, with magnetic resonance being the most indicated imaging technique. In cases of superficial lesions, the use of ultrasound techniques may be of interest.

The differential diagnosis should mainly include other vascular pathologies, such as haemangiomas or blood vessel malformations. At intraoral level, benign epithelial lesions, such as squamous papillomas, may occasionally be included (5).

The clinical management of lymphatic malformations will depend on the extension and location of the lesion, as well as the patient’s age. Two basic types of treatment have been described for the management of vascular malformations, which are surgical treatment and the use of different sclerosing agents (6).

The aim of this article is to describe the clinical and histopathological characteristics of a diagnosed case of oral lymphatic malformation, as well as to describe its surgical management.

## Case Report

20-year-old male, who attended the clinic for assessment of an asymptomatic lesion on the vestibular gingiva detected during dental examination. The patient did not recognise any traumatic factors associated with the appearance of the lesion. Since he became aware of the appearance of the lesion, he indicated no significant changes.

The patient had no medical history of interest, nor did report any known allergies. He indicated that he was a non-smoker and occasional alcohol consumer.

**Figure 1:**
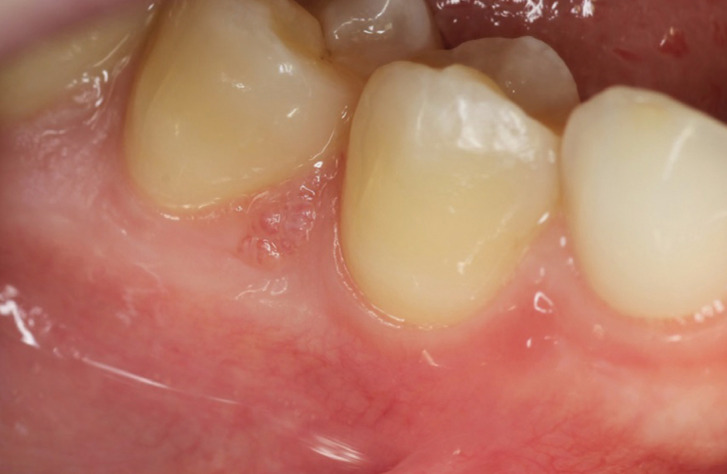
Clinical appearance of the lymphatic malformation. An elevated lesion consisting of multiple vesicles with a translucent and slightly erythematous colouration is recognisable.

Clinical examination revealed a lesion showing multiple exophytic vesicles, located in the attached vestibular gingiva, at the level of the papilla between 4.4 and 4.5. The lesion was partially pedunculated presenting irregular surface and slightly erythematous colour (Fig. 1). On palpation it was asymptomatic and non-bleeding. Periodontal examination healthy.

With all these data, lymphatic malformation was our presumptive clinical diagnosis, including localized juvenile spongiotic gingival hyperplasia in the differential diagnosis.

**Figure 2:**
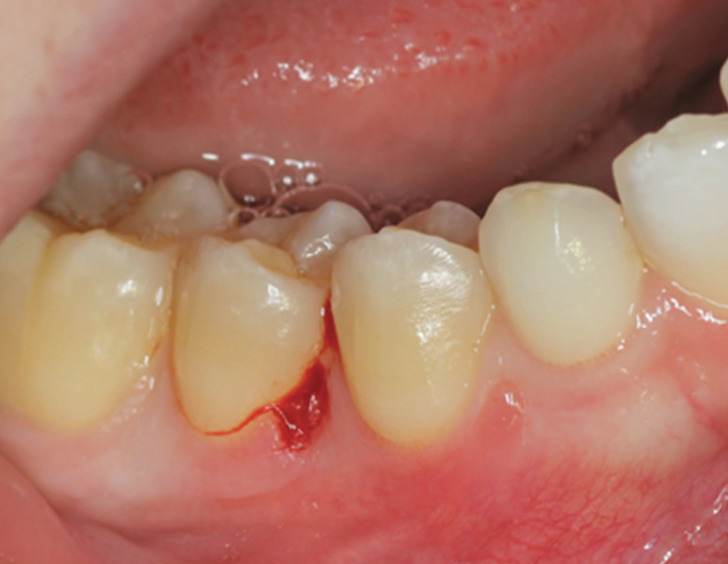
Surgical bed after lesion removal.

Given the clinical characteristics of the lesion, it was decided to perform its removal and subsequent histopathological analysis. Under infiltrative anaesthesia using 4% articaine with 1:100.000 adrenaline and with a No. 3 scalpel and No. 15 blade, surgical exeresis was performed, leaving the surgical bed healing by secondary intention (Fig. 2).

**Figure 3:**
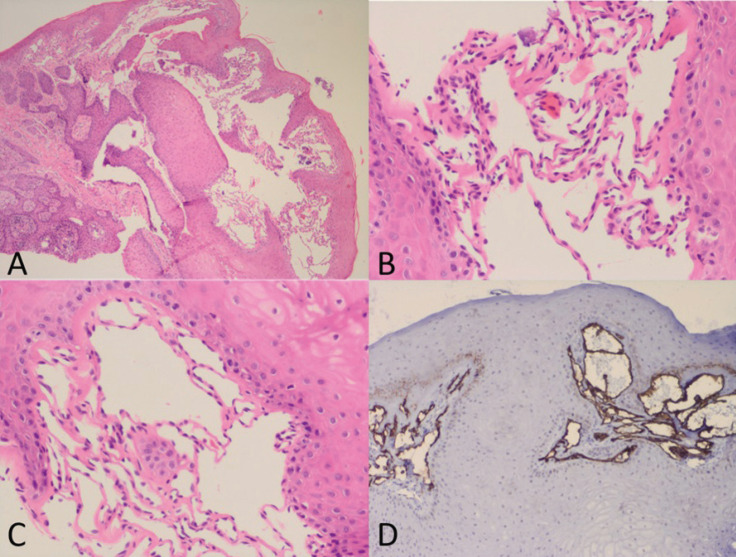
A) Multiple subepithelial lymphatic vascular structures (H&E. 20x) B-C) Detail of the thin endothelium that presents the lymphatic vascular malformation and the absence of erythrocytes inside (H&E. 40x). D) D2-40 immunohistochemical marker showing positivity in lymphatic endothelial cells (D2-40. 40x).

Histopathologic study revealed a subepithelial vascular lesion consisting of multiple small vessels and thin endothelium (Fig. 3A). These vessels had a lymphatic appearance, and were found anastomosed with no haematic content and with valvular elements inside them (Fig. 3. B,C). On the basis of these data, a histopathological diagnosis of microcystic lymphatic malformation was established. An immunohistochemical study with D2-40 marker was performed, which corroborated the lymphatic origin of the vascular malformation (Fig. 3. D).

**Figure 4:**
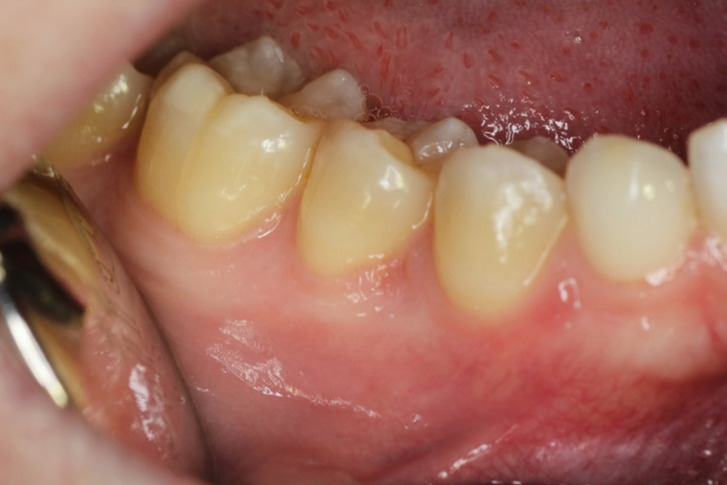
Clinical appearance 7 days after surgery.

At the 7 days follow-up, adequate healing was observed, with no functional alterations (Fig. 4). No recurrence of the lesion has been noted at 6 months follow up.

## Discussion

The case presented shows the clinical and histopathological expression of a microcystic lymphatic malformation in attached gingiva. Even though the gingiva is not the typical location of LM in the oral cavity, it has been reported to constitute about 5% of documented cases (5).

Although the actual prevalence of lymphatic oral malformations is not known, the diagnosis of this condition is between 0.02 and 0.16% of the biopsies analysed in oral pathology laboratories (5).

In recent years, several molecular alterations related to the appearance of lymphatic malformations, both sporadic and those involved in genetic syndromes, have been reported (7,8). The main one is associated with the protein mammalian target of rapamycin, also known as mTOR (8). It has been described that this protein or some of its components are overactivated in cases of both malformative and neoplastic lymphatic alterations (8). This fact is important, as it is generating changes in the management of lymphatic malformations. There are drugs, such as silorimus, that block the expression of mTOR protein and their infiltration is beginning to be used in the management of LM with very favourable results (9,10). Specifically in the case of oral lymphatic malformations, a recent study by Gomes *et al*., published in 2022, describes the activation of PI3K/AKT and MAPK/ERK intracellular pathways (7).

Meirelles *et al*., indicated that only 30.3% of the cases of oral lymphatic malformation had a clinical diagnosis of this entity (5). This fact makes it necessary to promote knowledge of the clinical manifestations of LM, so that the dentist has the capacity and resources to make an appropriate diagnosis of this entity. Haemangiomas or blood vessel malformations and squamous papillomas are among the main clinical conditions that are confused with LM (5). In the case of blood vessel malformations, both entities can be easily confused, since in some cases LM undergo a process of blood extravasation due to a traumatic component. In the case of squamous papillomas, as happens in our case, due to the irregular surface of the lesions, which may suggest an epithelial origin. In our case, we did not include squamous papilloma in the differential diagnosis, as these lesions, when they affect keratinized mucosa, have a whitish colour, due to the greater superficial keratinization presented by squamous papilloma lesions. In contrast, we did include localised juvenile spongiotic gingival hyperplasia in the differential diagnosis, since it is an entity that exclusively affects keratinized gingiva, presenting an irregular surface (11-13), as shown in our case (Fig. 1). Furthermore, it mainly affects patients in the second decade of life (11-13), similar to the case presented. Although it is true that the differential aspect could be the fact that localised juvenile spongiotic gingival hyperplasia lesions are characteristically erythematous, whereas in our case the colour was almost similar to the rest of the oral mucosa.

In any case, therapeutic management is similar, albeit the histopathological characteristics are what would help differentiate both entities.

LM can acquire very different sizes, although most intraoral lesions are smaller than 1cm (5), as in the case we illustrate.

In relation to the clinical management of the case, in our case, due to the size and location, we decided to proceed with surgical removal and its subsequent histopathological study, which is the treatment of choice for small and superficial lesions. On the other hand, in larger lesions and where there may be a post-surgical functional problem, treatment with sclerosing agents may be considered, including bleomycin, OK-432, alcohol, polidocanol or others (14,15).

In conclusion, we may indicate that lymphatic malformations are a rare pathology of the oral mucosa that can be expressed by the presence of multiple vesicular elements of translucent or reddish colouring and should be included in the differential diagnosis of vascular lesions. It is important to know the characteristics of this oral pathology in order to establish a proper diagnosis and clinical management.
